# Sirtuin 1 Facilitates Generation of Induced Pluripotent Stem Cells from Mouse Embryonic Fibroblasts through the miR-34a and p53 Pathways

**DOI:** 10.1371/journal.pone.0045633

**Published:** 2012-09-21

**Authors:** Yin Lau Lee, Qian Peng, Sze Wan Fong, Andy C. H. Chen, Kai Fai Lee, Ernest H. Y. Ng, Andras Nagy, William S. B. Yeung

**Affiliations:** 1 Department of Obstetrics and Gynaecology, The University of Hong Kong, Hong Kong, China; 2 Department of Obstetrics and Gynecology, University of Toronto, Toronto, Canada; 3 Samuel Lunenfeld Research Institute, Mount Sinai Hospital, Toronto, Canada; Indian Institute of Toxicology Reserach, India

## Abstract

Forced-expression of transcription factors can reprogram somatic cells into induced pluripotent stem cells (iPSC). Recent studies show that the reprogramming efficiency can be improved by inclusion of small molecules that regulate chromatin modifying enzymes. We report here that sirtuin 1 (SIRT1), a member of the sirtuin family of NAD^+^-dependent protein deacetylases, is involved in iPSC formation. By using an efficient mouse secondary fibroblast reprogramming system with doxycycline (DOX) inducible Yamanaka’s transcription factors delivered by piggyBac (PB) transposition (2°F/1B MEF), we show that SIRT1 knockdown decreased while resveratrol (RSV) increased the efficiency of iPSC formation. The treatments were associated with altered acetylated p53 and its downstream *Nanog* but not *p21* expression. The stimulatory effect was also confirmed by SIRT1 over-expression, which stimulated the formation of colonies with induced *Nanog* and reduced *p21* expression. Furthermore, the effects of RSV and SIRT1 knockdown on reprogramming were most pronounced during the initiation phase of reprogramming. MicroRNA-34a is a known regulator of SIRT1. Its inhibitor increased, while its mimics reduced iPSC formation. The stimulatory effect of SIRT1 during reprogramming was also confirmed in the primary MEF. RSV increased while tenovin-6, a small molecule that activates p53 through SIRT1 inhibition, suppressed reprogramming. In conclusion, SIRT1 enhances iPSC generation, in part, through deacetylation of p53, inhibition of p21 and enhancement of Nanog expression.

## Introduction

Reprogramming of adult somatic cells into induced pluripotent stem cells (iPSC) is one of the most significant scientific breakthroughs in recent years. iPSCs were first generated from mouse fibroblasts by the introduction of 4 transcriptional factors (Yamanaka’s factors), c-Myc, Klf4, Oct4 and Sox2 (MKOS) using retroviral system [1]. The same four factors were subsequently reported to reprogram fibroblasts from human [2], monkey [3], pig [4], rabbit [5] and horse [6], suggesting a conserved reprogramming mechanism in different species. Functionally, mouse iPSCs can produce chimeric mice, contribute to germline transmission, and most importantly generate “all iPSCs” animals [7]–[9]. These observations demonstrate that iPSC technology can potentially be used to generate patient-specific stem cells for regenerative medicine and to develop a model for studying disease processes using iPSCs generated from the patient of interest.

Small molecules that remodel chromatin and alter gene expression increase the reprogramming efficiency in iPSC production. For instance, valporic acid (VPA), a class I and II histone deacetylase (HDAC) inhibitor promotes the generation of mouse and human iPSCs [10] possibly by enhancing the Oct4 promotor activity [11]. Another HDAC inhibitor, butyrate also enhances iPSC generation by increasing acetylation of histone H3 and demethylation of promoter of pluripotency-related genes [12], [13]. Thus, chromatin modification is an important step in reprogramming.

Sirtuin 1 is a member of the sirtuin family of NAD^+^-dependent protein deacetylases. It is a class III HDAC and does not respond to inhibitors of Class I, II, and IV HDACs [14]. It is normally associated with transcriptional silencing through modulating chromatin function by direct deacetylation of histones and promoting alterations in the methylation of histones and DNA. The latter is accomplished by recruiting histone methylation or DNA CpG methylation enzymes to chromatin. In addition, the enzyme can directly interact and deacetylate a number of transcription factors and coregulators, leading to the positive and negative regulation of target gene expression (see review in reference [15]). In mouse ESCs (mESC), SIRT1 blocks nuclear translocation of p53 and inhibits p53-mediated suppression of Nanog expression [16]. Differentiation of human ESCs (hESC) causes down-regulation of SIRT1 and reactivation of key developmental genes that are epigenetically repressed by the histone deacetylase activity of SIRT1 [17]. Whether SIRT1 is involved in reprogramming by reversion of the above processes is not known.

MicroRNAs (miRNAs) are small non-coding RNAs. ESCs lacking miRNA biogenesis protein were defective in proliferation and differentiation [18], [19]. The reprogramming efficiency of mouse iPSCs was enhanced by miRNAs found in ESCs [20], [21]. Recently, several miRNAs including miR-181a and b, miR-9, miR-204, miR-199b, and miR-135a were shown to down-regulate SIRT1 expression in mESC [22].

We hypothesize that SIRT1 functions as a positive epigenetic regulator in the maintenance of hESC and the reprogramming of fibroblasts to iPSCs. In this study, we investigated the roles of SIRT1 in reprogramming, and found that it stimulated iPSC formation through the miR-34a-SIRT1-p53 pathway.

## Experimental Procedures

### Human Embryonic Stem Cell Culture and Differentiation

The hESC line H9 (WiCell Research Institute, Madison, WI) was maintained in mitomycin C inactivated human foreskin fibroblast (hFF-1, ATCC, Manassas, VA) using VitroHes (Vitrolife, Göteborg, Sweden) supplemented with 20 ng/ml bFGF (Invitrogen, Life Technologies, NY, U.S.A) and passaged by mechanical expansion. For feeder-free experiment, H9 was enzymatically digested with 1 mg/ml collagenase type IV and cultured in geltrex matrix coated plates with StemPro (Invitrogen) medium supplemented with 0.1 mM 2-mercaptoethanol and 10 ng/ml bFGF. To induce differentiation, the medium was supplemented with retinoic acid (RA) at a concentration of 5 µM (Sigma, St. Louis, MO) or bone morphogenetic protein 4 (BMP4, R&D Systems, Minneapolis, MN) at 10 ng/ml in the absence of bFGF 24 hours after seeding, and cultured for 8 days. H9 cultured in bFGF supplemented medium for 8 days was used as the control. H9 was differentiated into embryoid bodies (EB) as described [23] with some modifications. Briefly, mechanically dissected H9 fragments was centrifuged at 450 g for 5 minutes and allowed to aggregate in round-bottom low attachment 96-well plate (Nunc, Kamstrupvej, Roskilde) for 4 days, before transferred to gelatin coated plate for further attachment growth for 25 days.

### Mouse Embryonic Stem Cell Culture and Differentiation

Mouse embryonic stem cells (L4) were obtained from the Transgenic Core Facility, Department of Biochemistry, The University of Hong Kong. L4 and miPSC were cultured in mESC medium [DMEM with high glucose, 100 units/ml penicillin and 100 µg/ml streptomycin (Gibco, Life Technologies), 0.1 mM MEM non-essential amino-acids (Gibco), sodium pyruvate (110 mg/L, Gibco), 50 µM beta-mercaptoethanol, 15% FBS (Gibco) and 1000 units/ml LIF (Millipore). L4 or miPSC were differentiated into embryoid bodies using hanging drop method for the first 2 days followed by 3 days of suspension culture in ultra-low attachment 96 well plate. The EB was then allowed to attach to gelatin-coated plate for further culture.

### Secondary Mouse Embryonic Fibroblast System, iPSC Induction and Culture

Secondary PB-iPSC-derived mouse embryonic fibroblasts (2°F/1B MEF) containing the doxycycline (DOX) inducible MKOS reprogramming factors and wild type C57BL/6 MEF (wt-MEF) were isolated as described in [24]. 2°F/1B MEF was seeded at 833 cells/cm^2^ together with wt-MEF. MKOS was induced with 1.5 µg/ml DOX (Sigma) in mESC medium the following day after seeding. The iPSC colonies were assessed on day 10, day 15 and day 21 as described in each experiment.

### Lentivirus Packaging and Primary Mouse Embryonic Fibroblast Transduction and Reprogramming

Primary MEF were obtained from ICR mice at 14.5 dpc. Reprogramming was performed by using lentiviruses produced by TetO-FUW-mOSKM (Addgene #20321) containing doxycycline inducible MKOS reprogramming factors cDNAs in a polycistronic viral vector. 293T cells were transfected with FUW-M2rtTA (Addgene # 20342) and TetO-FUW-mOSKM (Addgene #20321) accompanied with pLP1, pLP2, and pLP/VSVG plasmids (Invitrogen) using lipofectamine 2000 (Invitrogen). Viral supernatant were harvested at 48 and 72 hours after transfection. MEFs were infected with the lentiviruses for 24 hours before exchanged of regular MEF medium. After three days, cells were split onto gelatin coated plates. Twenty four hours after seeding, the cells were treated with 1.5 µg/ml DOX in mESC medium for induction of the Yamanaka’s factors.

### Quantitative PCR and Western Blotting

Total RNAs (large and small RNA) were extracted from the total cells or iPSC colonies by the *mir*Vana™ miRNA isolation Kit (Ambion, Life Technologies) following the manufacturer’s protocol and subjected to reverse transcription using TaqMan® Reverse Transcription Reagents or TaqMan® MicroRNA reverse transcription kit (Applied Biosystems Inc., Life Technologies). Real time quantitative PCR (qPCR) was performed using the Applied Biosystems 7500 Real-Time PCR System for the quantification of mRNA by TaqMan® Gene Expression Assays. The detection of human or mouse Nanog, Sirt1, p21, Snail2 and Cdh1 mRNA was normalized with the endogenous 18S ribosomal RNA using the 2^−ΔΔC^_T_ method for quantification. The resulting data were analyzed by the software provided by the manufacturer (Applied Biosystems Inc.). For immunoblotting, the cells were lysed in cell lysis buffer (Ambion) containing protease inhibitors (Calbiochem, Darmstadt, Germany). Equal amount of protein from each sample was heat inactivated and separated by electrophoresis on 10% SDS-PAGE and transferred to polyvinylidene fluoride membranes (PVDF; Immobilon-P, Millipore, Billerica, MA, U.S.A). The membranes were blotted with antibodies against SIRT1 (Santa Cruz, CA, U.S.A), OCT4 (Santa Cruz), PCNA (Dako, Denmark), acetylated p53 (CST, Cell Signaling Technology, Danvers, MA), p53 (CST) and β-actin (Sigma) followed by appropriate horseradish peroxidase-conjugated secondary antibodies and developed by enhanced chemiluminescence (Westsave Up, Abfrontier Co. Ltd, Korea).

### Alkaline Phosphatase Activity and Immunocytochemistry

The cells were fixed with 4% paraformaldehyde and washed with PBST. The alkaline phosphatase activity was determined by the ES Cell Characterization Kit (Chemicon, Billerica, MA) following the manufacturer’s protocol. Colonies stained red indicated positive alkaline phosphatase activity. The expression of mouse pluripotent markers, SSEA-1 and NANOG was examined by immunocytochemical staining. The iPSC colonies 15 days post-DOX treatment were fixed with 4% paraformaldehyde and permeablized with 0.1% Triton before incubation with antibody against SSEA-1 (Chemicon) and NANOG (R&D Systems). The fluorescent images were observed under a confocal microscope (LSM 700, Carl Zeiss AG, Oberkochen, Germany).

### siRNA, SIRT1 Plasmid and miRNA Transfection

2°F/1B MEF were seeded at 1666 cells/cm^2^ without wt-MEF and transfected with 100 nM siRNAs (scramble siRNA-A&B and SIRT1 siRNA, Santa Cruz) with lipofectamine 2000 (Invitrogen) 24 h after seeding. The over-expression of miR-34a was performed by transfection of 80 nM of Pre-miR™ miRNA precursor molecule of miR-34a (miR-34a precursor) or control precursor (Ambion) while knockdown of miRNA was performed by transfection of 80 nM miRCURY LNA™ miRNA knockdown probe, miR-34a inhibitor or control inhibitor (Exiqon, Vedbaek, Denmark) according to our established protocol [25]. SIRT1 plasmid pCruzHA SIRT1 (Addgene Plasmid# 10962) were kindly provided by Dr. Toren Finkel. To over-express SIRT1, pCruzHA SIRT1 was amplified and 4, 8 or 16 ng/ml of the plasmid was transfected with lipofectamine 2000. After transfection, the cells were fed with mESC medium supplemented with DOX thereafter.

### RSV and Tenovin-6 Treatments

For RSV treatment, the 2°F/1B MEF was seeded at 833 cells/cm^2^ together with wt-MEF and treated with different concentrations of RSV (Sigma) in the presence of DOX. For tenovin-6 (Santa Cruz) treatment, OSKM transduced primary MEF were seeded at 1666 cells/cm^2^ and treated with 1 and 5 µM of tenovin-6 together with DOX. The media were changed every other day.

### Proliferation Assay

The proliferation of MEF after transfection of different concentration of SIRT1 plasmids was performed by The CyQUANT® NF Cell Proliferation Assay Kit (Invitrogen) according to the manufacturer instructions. The cells were stained and the fluorescence was read using excitation at 485 nm and emission at 530 nm using a plate reader (Tecan Infinite F200, San Jose, CA).

### Statistical Analysis

Data were analyzed and plotted using SigmaPlot software (Aspire Software International, Leesburg, VA, USA). Statistical analysis was performed by t-test, Rank Sum test and One Way ANOVA as appropriate. Significant differences between groups was considered when p<0.05.

## Results

### SIRT1 is Down-regulated in Differentiated Human ESCs

**Figure 1:**
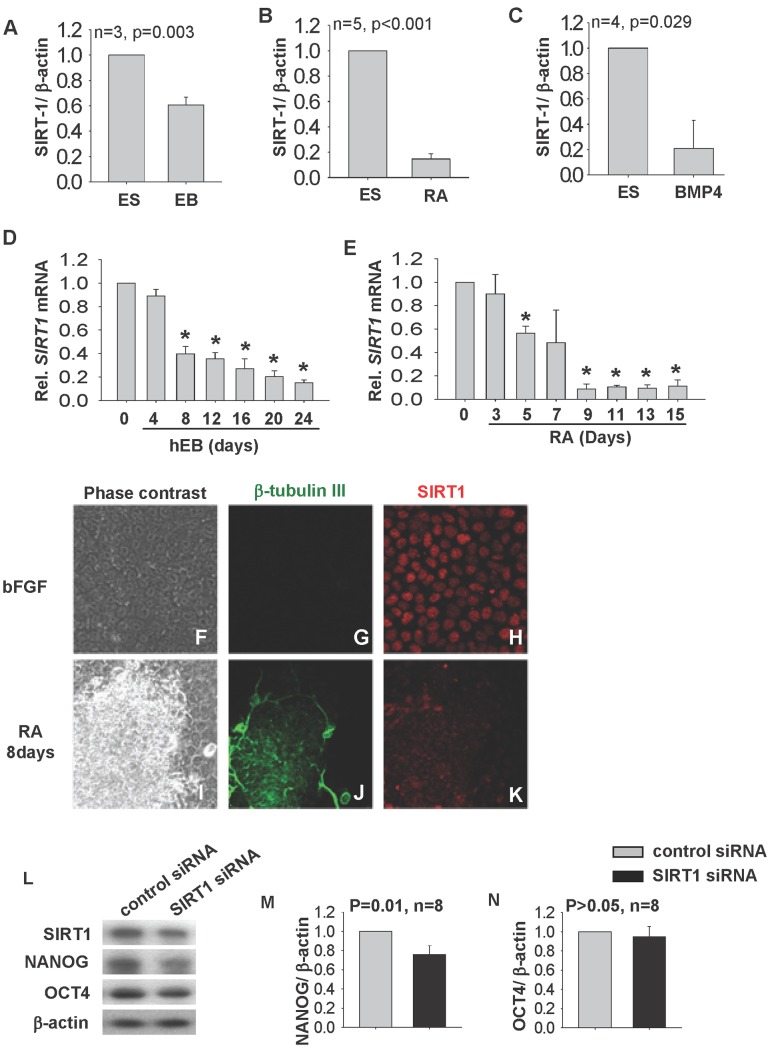
SIRT1 expression during hESC differentiation. Relative SIRT1 protein levels in undifferentiated H9 cultured in bFGF (ES) for 6 days or differentiated to EB in differentiating medium for 25 days (A), after RA (B) and BMP4 (C) induced differentiation. Results are shown as the relative amount of SIRT1 to β-actin levels. (D, E) Time dependent SIRT1 mRNA expressions in EB from Day 4 to Day 24 and after RA treatment for 3 to 15 days. The expression levels were relative to undifferentiated H9 (D0). *p<0.05 when compared to D0 control. Confocal images showing localization of SIRT1 (red) and ectoderm marker β-tubulin III (green) in bFGF (F, G, H) or RA treated (I, J, K) H9 cells. Relative NANOG and OCT4 protein expression in H9 after transfected with control-siRNA or SIRT1-siRNA and normalized with internal control, β-actin. Representative diagram of Western blotting of SIRT1, NANOG and OCT4 proteins was shown (L, M, N). p-value, Rank Sum Test.

Human embryonic stem cell line H9 was either cultured in bFGF for 6 days or differentiated into EB for 25 days. The proteins were subjected to Western blot analysis and the relative SIRT1 expression level was normalized with that of β-actin. The expression of SIRT1 was high in H9 but was down-regulated to 50% of the undifferentiated level in EBs (Fig. 1A). To confirm if the down-regulation of SIRT1 during differentiation is lineage specific, we used RA and BMP4 to induce the differentiation of epithelial cells [26] and trophoblast cells [27] respectively. Both RA and BMP4 treatments significantly suppressed *OCT4* and *NANOG* mRNA expressions (Fig. S1A, B). While RA induced the expression of epithelial marker, *TP63* (Fig. S1A), BMP4 induced the expression of trophoblast marker, cytokeratin 7 (*KRT7*, Fig. S1B). Upon induced differentiation with RA (Fig. 1B) or BMP4 (Fig. 1C) for 8 days, SIRT1 protein levels were significantly decreased to 20% of the undifferentiated cells. We then set out to study the temporal expressions of SIRT1 during EB formation and 15 days of RA treatments. It was found that *SIRT1* mRNA dropped drastically in day 8 EB and time-dependently decreased from day 12 to day 24 (Fig. 1D). RA also suppressed *SIRT1* expression time-dependently, which started from day 5 and progressively thereafter (Fig. 1E). In both differentiation protocols, the temporal expressions of *SIRT1* mRNA were positively associated with *NANOG* (Fig. S1F, I) and *OCT4* (Fig. S1G, J) but negatively correlated with the 3 germ layers markers, *AMY* (endoderm, Fig. S1C), *REN* (mesoderm, Fig. S1D) and *NEFH* (ectoderm, Fig. S1E) during EB formation and *TP63* upon RA treatment (Fig. S1H). RA treatment induced the expression of the neuronal marker, β-tubulin III (Fig. 1J). Consistently, nuclear SIRT1 immunoreactivity, which was strong in the undifferentiated H9 cells (Fig. 1H), was diminished after 8 days of RA-induced differentiation (Fig. 1K).

To further determine the relationship between SIRT1 and expression of pluripotent markers in hESC, we used siRNA to knockdown SIRT1 expression. As shown by the Western blotting analysis (Figure 1L), the treatment suppressed the expression of SIRT1 protein (∼30%, Fig. 1L) and decreased the mRNA (50%, p = 0.031, Fig. S1K) and protein expression of the pluripotent marker, NANOG (30%, p = 0.01, Fig. 1M), but not that of OCT4 (Fig. S1L and Fig. 1N).

### SIRT1 is Up-regulated during Mouse iPSC Formation

To test whether SIRT1 could enhance reprogramming, we used the secondary mouse fibroblasts reprogramming system efficiently returning to iPSC state using DOX inducible MKOS transcription factors delivered by piggyBac (PB) transposons. To generate such secondary fibroblasts, primary iPSCs were aggregated with 2.5 dpc embryos to produce iPSCs chimaeras, which were then used to derive “chimeric” secondary mouse embryonic fibroblasts (2°F/1B MEF) [24]. Upon DOX activation of the MKOS transgenes, we obtained iPSC like colonies that expressed the mESC pluripotent cell marker SSEA-1 and NANOG immnoreactivities (Fig. S2). These colonies were all GFP positive, consistent with their generation from iPSCs with constitutive GFP expression [24].

**Figure 2:**
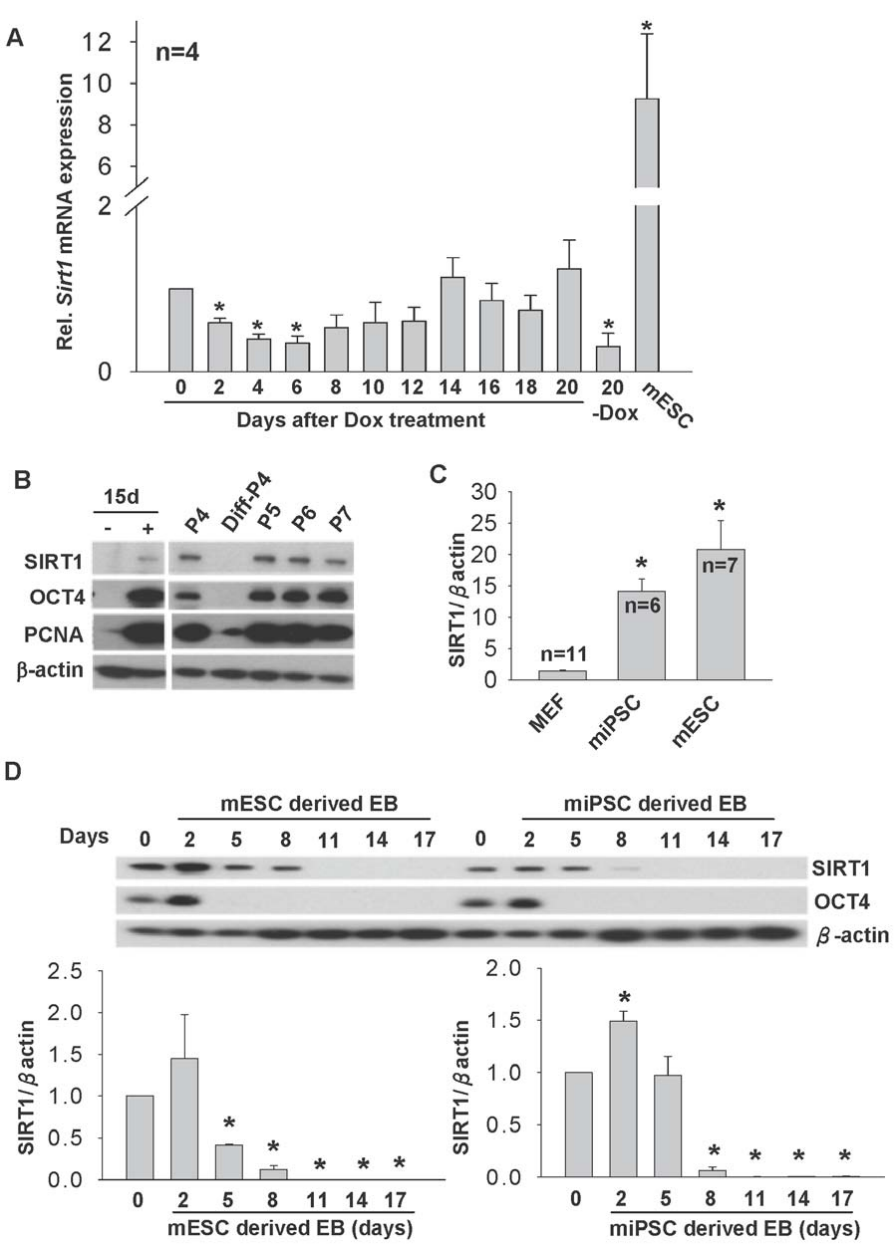
SIRT1 expression during iPSC formation and differentiation of ESCs and iPSCs in mouse model. (A) Temporal expression of *Sirt1* mRNA on day 0 to day 20 after DOX treatment. MEF without DOX on day 20 (20-DOX) and mESC were included. (B) Western blotting analysis of SIRT1, OCT4 and PCNA in 2°F/1B MEF without (-DOX) and with (+DOX) DOX treatment for 15 days, serially passaged iPSC from passages 4 (P4), 5–7 (P5, P6, P7) and differentiated colonies at passage 4 (Diff-P4). (C) The relative expression levels of SIRT1 protein in MEF, miPSC and mESC. (D) Relative SIRT1 protein expressions in embryoid bodies collected from mESC and miPSC on days 2, 5, 8, 11, 14 and 17 after differentiation. D0 are the undifferentiated cell control. *p<0.05 when compared to D0 control. Representative diagrams of Western Blotting of SIRT1 and OCT4 during embryoid body formation were shown.

We set out to study the expression of SIRT1 during reprogramming of 2°F/1B MEF. We first determined the temporal change in the expression of *Sirt1* during a 20 days’ reprogramming period. *Sirt1* mRNA was significantly reduced upon DOX treatment for the first 6 days, and increased progressively thereafter. After 20 days’ of reprogramming with DOX, *Sirt1* mRNA was much higher than those cells without DOX treatment (-DOX). However, the expression level was far less than in mESC (Fig. 2A). We then collected 2°F/1B MEF cultured with or without DOX treatment for 15 days and subjected to Western blotting analysis. Consistent with previous findings [17], SIRT1 protein was undetectable or barely detected in MEF. However, a faint band of SIRT1 was detected upon DOX treatment, and associated with high expressions of OCT4 and proliferating cell nuclear antigen (PCNA) proteins (Fig. 2B, left). The iPSC like colonies were picked and serially passaged, SIRT1 signal was enhanced and highly expressed in undifferentiated, but not in differentiated iPSC colonies, indicated that SIRT1 expression was increased with passages in iPSCs after reprogramming (Fig. 2B, right). We also compared the protein levels of SIRT1 in MEF, serially passaged miPSCs and mESCs. The highest SIRT1 protein level was found in mESCs followed by miPSCs. The level of SIRT1 in MEF was significantly lower than mESCs and miPSCs (Fig. 2C). In addition, mESC and serially passaged iPSC were subjected to EB formation and the samples were collected on days 2, 5, 8, 11, 14 and 17. Similar to mESCs (Fig. 2D, left), SIRT1 protein was down-regulated upon differentiation of iPSCs to EBs in a time dependent manner (Fig. 2D, right).

### Knockdown of SIRT1 Suppresses but Resveratrol Enhances iPSC Formation

**Figure 3:**
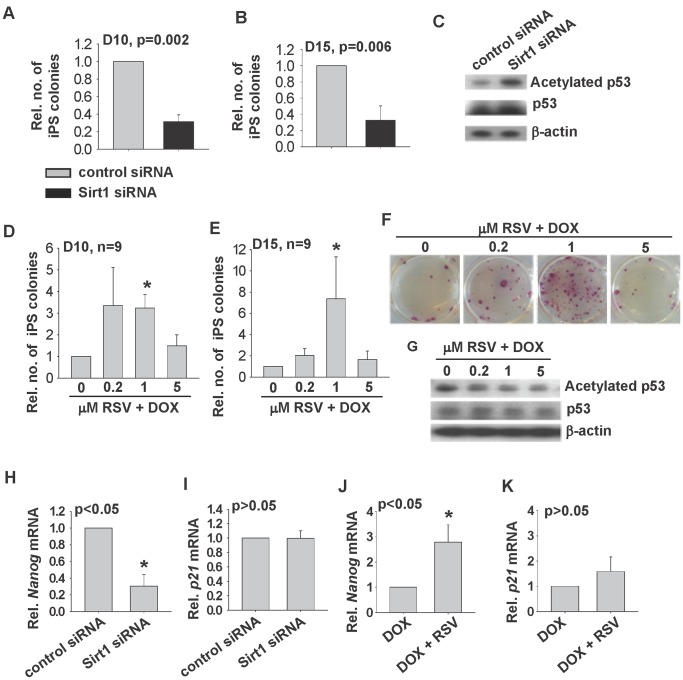
Effects of SIRT1-siRNA and RSV on iPSC formation. The relative number of DOX induced iPSC colonies formed on Day 10 (A) and Day 15 (B) after transfection with control-siRNA or SIRT1-siRNA. The percentage shown was relative to the control groups (n = 6). (C) Western blotting analysis of acetylated p53 and p53 in Day 15 iPSC colonies was shown. The relative number of iPSC colonies on Day 10 (D) and Day 15 (E) formed upon treatment with 0.2, 1 and 5 µM RSV (n = 9). Representative alkaline phosphatase staining (F) and Western blotting analysis of acetylated p53 and p53 of Day 15 iPSC colonies was shown (G). *p<0.01 when compared to DOX treatment alone. The relative *Nanog* and *p21* mRNA expressions in Day 15 iPSC colonies after treatment with siRNA (H, I) or RSV (J, K).

The role of SIRT1 during reprogramming was first studied by transfection of Sirt1 siRNA or control siRNA to 2°F/1B MEF followed by DOX induction. The iPSC colonies were counted on day 10 and day 15 after DOX induction. The results showed that the colony number formed after treatment with Sirt1 siRNA was three fold lower than that of the control group (p = 0.002 and p = 0.006 respectively) (Fig. 3A and B). The colonies were collected on day 15 and subjected to Western Blotting analysis of the acetylated p53 level. As expected, SIRT1 knockdown increased the level of acetylated p53 protein by 20% (Fig. 3C).

We also used a reported SIRT1 activator, resveratrol (RSV) [28] to treat 2°F/1B MEF during reprogramming. 2°F/1B MEF was treated with 0.2, 1 or 5 µM of RSV in the presence of DOX. RSV at concentrations of 0.2 and 1 µM increased the number of colonies formed from 2°F/1B MEF on both day 10 and day 15 (Fig. 3D and E). The strongest effect was observed with the use of 1 µM of RSV. It resulted in a 6 fold increase in the iPSC colony formation on day 15, which was significantly (p<0.05) higher than that of the control (Fig. 3E and F). Higher concentration of RSV (5 µM) had no effect on the reprogramming efficiency. Western Blotting analysis also showed that RSV decreased the acetylated p53 level in the day 15 iPSC colonies (Fig. 3G). The siRNA and RSV treated colonies were collected and subjected to qPCR analysis of *Nanog* and *p21* mRNA expression. The results indicated that *Nanog* but not *p21* mRNA expression was significantly decreased in iPSC colonies by si-SIRT1 treatment (Fig. 3H and I) and increased in iPSC colonies by 1 µM RSV treatment (Fig. 3J and 3K).

**Figure 4:**
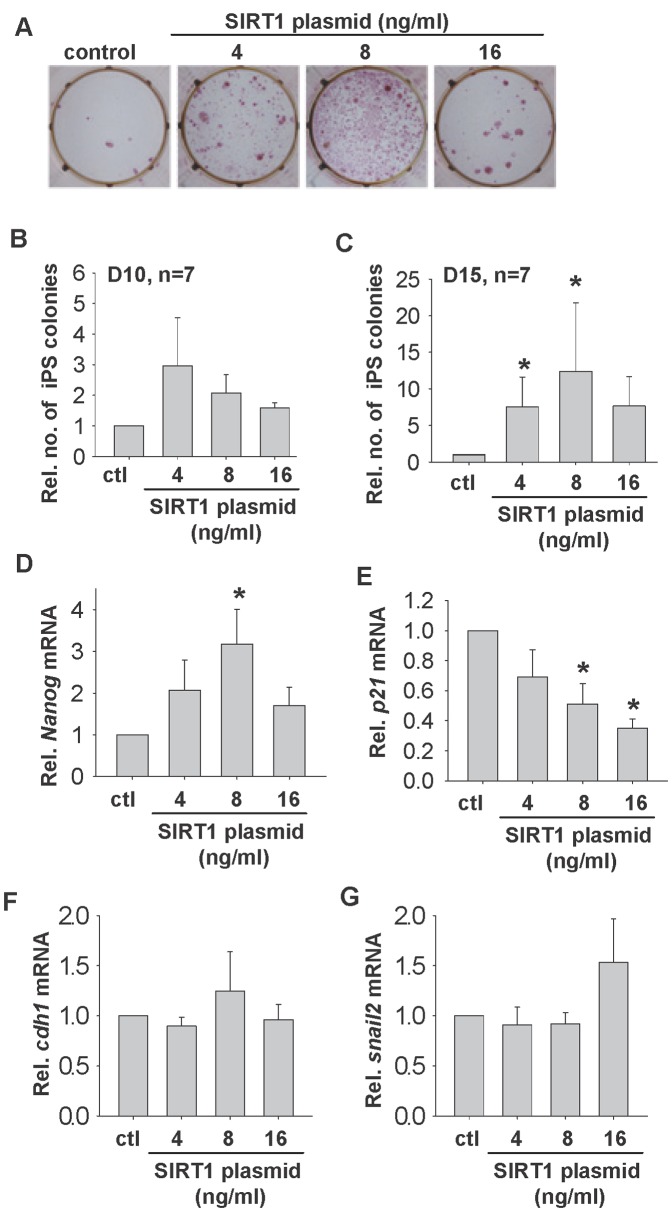
Effects of SIRT1 over-expression on reprogramming. Representative alkaline phosphatase staining of iPSC colonies on Day 15 (A) and the relative number of iPSC colonies formed upon treatment with 4, 8 and 16 ng/ml Sirt1 plasmid (n = 7) on Day 10 (B) and Day 15 (C). The relative *Nanog* (D) and *p21* (E), and MET markers, *cdh1* (F) and *snail2* (G) mRNA expressions in Day 15 iPSC colonies after treatment with 4, 8 and 16 ng/ml of Sirt1 plamids. * p<0.05 when compared to control group.

### SIRT1 Over-expression Stimulates iPSC Formation

To further confirm the specificity of SIRT1 effect on reprogramming, 2°F/1B MEF was transfected with 4, 8 and 16 ng/ml SIRT1 plasmid during reprogramming. SIRT1 over-expression was confirmed in the cells 3 days post-transfection (Fig. S3A), and the treatment did not affect cell proliferation (Fig. S3B). SIRT1 over-expression increased colony formation on both day 10 and day 15 dose-dependently (Fig. 4A–C), in which 8 ng/ml of plasmid increased 10–15 fold in the number of iPSC colonies formed on day 15 (Fig. 4C). In addition, the treated colonies expressed significantly more *Nanog* (Fig. 4D) but lower *p21* (Fig. 4E) mRNA expressions. SIRT1 over-expression had no effect on expressions of MET markers, *Cdh1* (Fig. 4F) and *Snail2* (Fig. 4G).

**Figure 5:**
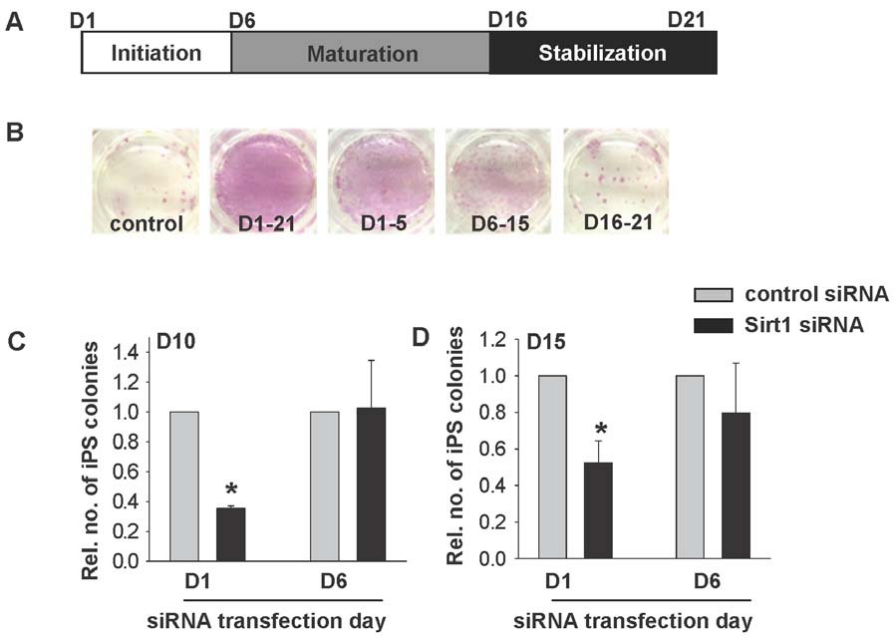
Temporal effect of SIRT1-siRNA and RSV on iPSC formation. (A) Schematic diagram showing the initiation (D1–5), maturation (D6–15) and stabilization (D16–21) phase of the reprogramming process. (B) Alkaline phosphatase stainings of iPSCs on day 21 post-DOX induction with RSV treatments during the reprogramming phases. The relative number of colonies formed on Day 10 (C) and Day 15 (D) after transfection with control siRNA or Sirt1 siRNA on Day 1 and Day 6 post-DOX treatment. *p<0.05 when compared to control group.

### RSV Acts on the Initiation Phase of Reprogramming

To define the period of action of RSV and si-SIRT1 on reprogramming of fibroblasts, 2°F/1B MEF was treated with DOX in the presence of 1 µM RSV at different phases of reprogramming, namely initiation phase (treatment covered day 1–5), maturation phase (treatment covered day 6–15), stabilization phase (treatment covered day 16–21) or whole reprogramming period (treatment covered day 1–21) (Fig. 5A). The colony were fixed on day 21 and the results showed that the action of RSV in producing more alkaline phosphatase positive colonies was most effective in the initiation phase (day 1–5) followed by the maturation phase (day 6–15). The number of colonies formed was highest when the treatment covered all the 21 days (Fig. 5B). To define the action of siRNA, 2°F/1B MEF was transfected with Sirt1 siRNA or control siRNA on day 1 or day 6 respectively during the reprogramming process. 2°F/1B MEF transfected with Sirt1 siRNA on day 1 of DOX induction (D1) formed significantly fewer iPSC colonies when compared to those transfected on day 6 post-DOX induction (D6) (Fig. 5C–D).

**Figure 6:**
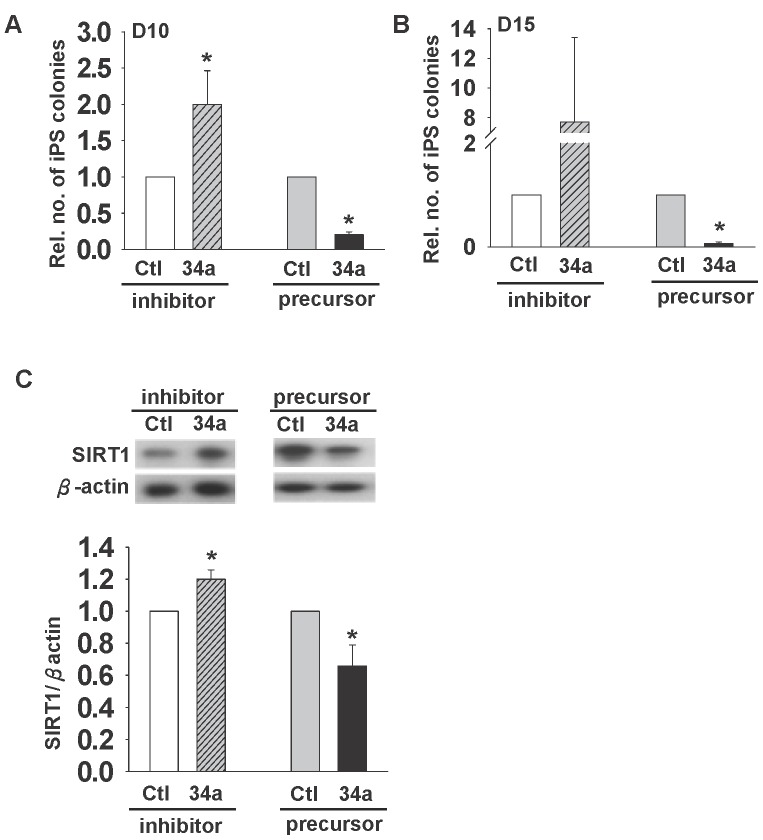
Effects of miR-34a on reprogramming. Relative number of colonies formed after transfection of miR-34a (34a) precursor and inhibitor on Day 10 (A) and 15 (**B**) post-DOX treatment when compared to the corresponding control (Ctl). (C) Relative SIRT1 protein expression upon transfection with miR-34a precursor and inhibitor after 72 h.

### miR-34a is Involved in iPSC Formation

Because SIRT1 has been reported to be a direct downstream target of miR-34a [29], the effects of miR-34a precursor and inhibitor on iPSC formation were then followed. 2°F/1B MEF was transfected with miR-34a precursor or inhibitor. The iPSC colonies formed were counted on day 10 and day 15, and compared to their respective controls. The results demonstrated that while miR-34a precursor inhibited the iPSC formation, miR-34a inhibitor increased the formation on day 10 (Fig. 6A) and day 15 (Fig. 6B). In addition, SIRT1 protein expression was significantly up-regulated and down-regulated by miR-34a inhibitor and miR-34a precursor (Fig. 6C), respectively, consistent with a role of miR-34a and SIRT1 in reprogramming.

**Figure 7:**
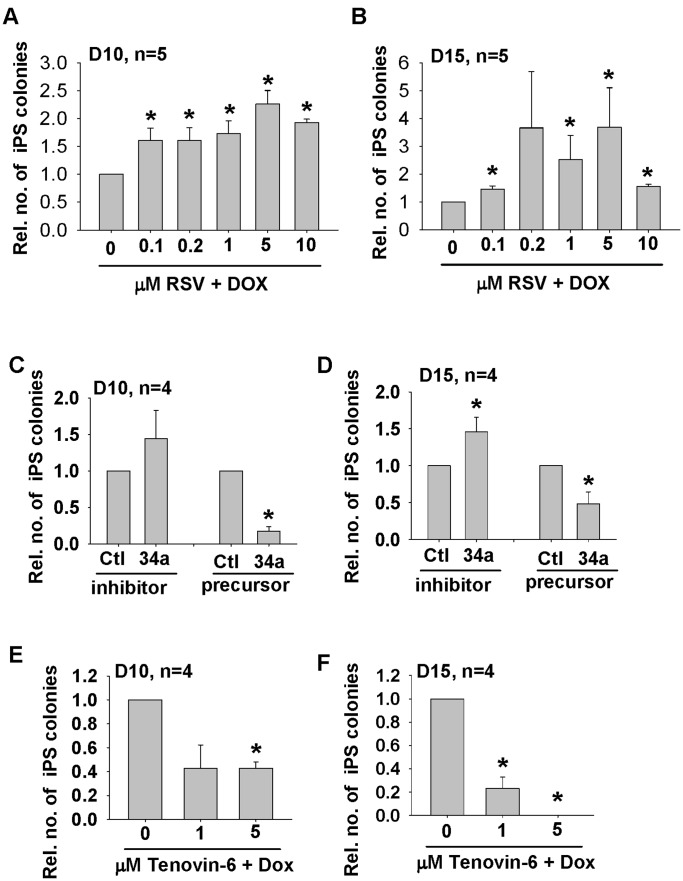
Effects of RSV, miR-34a and Tenovin-6 on primary MEF reprogramming. The relative number of iPSC colonies formed upon treatment with 0.1, 0.2, 1, 5 and 10 µM RSV (n = 5) on Day 10 (A) and Day 15 (B). *p<0.05 when compared to DOX treatment alone. Relative number of colonies formed after transfection of miR-34a (34a) precursor and inhibitor on Day 10 (C) and 15 (D) post-DOX treatment when compared to the corresponding control (Ctl). The relative number of iPSC colonies formed upon treatment with 1 and 5 µM tenovin-6 (n = 4) on Day 10 (E) and 15 (F). *p<0.05 when compared to DOX treatment alone.

### RSV Promotes and miR-34a Inhibits iPSC Formation in Primary MEF

Finally, to confirm if the effect of RSV and miR-34a on reprogramming was not restricted to piggybac transposon and secondary MEF, we examined primary MEFs transduced with MKOS-expressing lentivirus. We confirmed that RSV at concentrations of 0.1 to 10 µM stimulated a 3-fold increase in iPSC colony formation (Fig. 7A and B). Consistently, inhibitor of miR-34a stimulated while its mimics decreased colony formation (Fig. 7C and D). Besides, we also studied the effect of tenovin-6 that activated p53 through inhibition of the protein-deacetylating activities of SIRT1 [30] on reprogramming of the primary MEF. We treated the MEF with 1 and 5 µM tenovin-6 for 24 hours and found induction of both acetylated and total p53 (Figure S4). Interestingly, tenovin-6 dose-dependently decreased the colony formation (Fig. 7E and F), further supported the notion that the suppressive effect of SIRT1 on p53 is critical for reprogramming.

**Figure 8:**
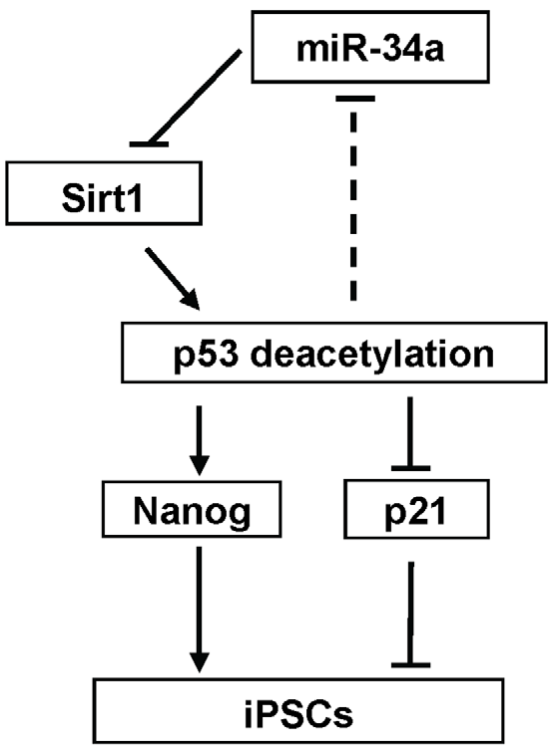
Schematic diagram showing the pathway of miR-34a-SIRT1-p53 during reprogramming.

## Discussion

In agreement with the reported finding of SIRT1 down-regulation in the hESC lines Shef-1 and H-181 during EB formation [17], we also found that SIRT1 was down-regulated in the H9 hESC line in a time dependent manner. Furthermore, our data extended the findings showing that SIRT1 expression was down-regulated during differentiation of hESC induced by RA and BMP4. Human ESCs treated with RA produces mainly ectoderm [26] while BMP4 treatment produces trophoblast [27], [31], primitive endoderm [32], [33] and mesoderm [34]. The correlation of the expression of SIRT1 and NANOG during differentiation and the SIRT1 knockdown-induced decrease in NANOG suggest that SIRT1 down-regulation is likely to be an early event common to differentiation of all cell lineages.

SIRT1 is a protein deacetylase normally associated with transcriptional silencing through histone deacetylation. It can modify histone proteins around the genes, thereby modulate gene expression epigenetically. Apart from acting as histone deacetylase, SIRT1 also deacetylates a number of non-histone proteins including p53 [35]. In tumor cells, SIRT1 deacetylates p53, leading to change in proliferation/apoptosis via the p53-p21 pathway [36]. In mouse ESCs, SIRT1 regulates apoptosis and Nanog expressions and protects the cells from oxidative stress via controlling p53 subcellular localization [16]. Interestingly, we demonstrated that SIRT1 knockdown led to lower NANOG but not OCT4 expression in hESCs, suggesting that SIRT1 may modulate the expression of NANOG through p53 deacetylation. SIRT1 inhibition increased p53 acetylation [37], leading to the transactivation of downstream targeting gene of p53, while the suppression of the p53 pathway has been reported to facilitate reprogramming [9]. In fact, our previous result also showed that p53 knockdown in secondary MEF enhanced iPSC formation [38]. We speculated that SIRT1 might be involved in the reprogramming process.

Due to the low efficiency in generating primary iPSCs, we adopted an efficient DOX inducible secondary PB-iPSC-derived mouse embryonic fibroblasts (2°F/1B MEF) [24] to study the role of SIRT1 in the entire reprogramming process. In contrast to its down-regulation during the differentiation of mESC and iPSCs, SIRT1 expression increased during reprogramming of mouse fibroblasts, though its level was much lower when compared to that of mESC. Comparable SIRT1 expression level to that of mESC was only attained in serially passaged iPSC, which is in line with the fact that extended passaging of iPSC resulted in enhanced pluripotency and diminished differential gene expression between ESCs and iPSCs [39]. The progressive increase of SIRT1 during passaging suggested that SIRT1 expression may be positively correlated with the pluripotency of the reprogrammed iPSCs, in which the partially reprogrammed iPSCs expressed low levels of SIRT1. High SIRT1 expression was only attained in fully reprogrammed cells. SIRT1 is an aging related gene and its expression decreased with increased population doublings and serial cell passages [40]. The significant drop of *Sirt1* mRNA in the 2°F/1B after 20 days of culture in the absence of DOX could be due to senescence of the MEFs. However, the increased expression of SIRT1 during reprogramming suggested that SIRT1 might be required for the iPSC formation.

Two observations support a role of SIRT1 as an enhancer in reprogramming. First, knockdown of SIRT1 at the onset of cellular reprogramming suppressed iPSC production by 3 folds. The effects of the suppression were more prominent on day 10 after DOX induction when compared to day 15, possibly due to the dilution effect of SIRT1 siRNA with cell proliferation. Second, treatment with RSV induced the formation of iPSCs. Recently, a paper was published concerning the positive effect of RSV on inducing iPSC formation [41], however the underlying mechanism is not reported. RSV is a plant polyphenol that can increase the protein expression of SIRT1 [42], and is regarded as a potent activator of SIRT1 activity [43]. To this end, although we did not find a significant increase in the *Sirt1* expression level after RSV treatment (data not shown), the stimulatory activity of RSV through SIRT1 was supported by the reduction of acetylated p53 level, a known substrate of SIRT1. The mechanism is further supported by analyzing the expression of the SIRT1 downstream targets in the p53 pathway [16], which showed that *Nanog* mRNA expression was significantly increased by RSV but decreased by SIRT1 siRNA treatments in iPSC colonies. These data are consistent with the notion that SIRT1 may alleviate the suppressive role of p53 on Nanog expression during reprogramming.

Apart from SIRT1, RSV has other targets. It reduces the activation of extracellular signal regulated kinases (ERK) in other cell types [44], [45]. Interestingly, inhibition of ERK promotes the formation of fully reprogrammed iPSCs [46]. Besides, our data showed that the iPSCs colony formation efficiency was lower when the cells were treated with 10 µM RSV than 5 µM RSV on day 15, indicating that higher concentration of RSV may affect other pathway(s) that inhibit reprogramming. In view of these possibilities, we over-expressed SIRT1 in 2°F/1B MEF to study the specific action of SIRT1 during reprogramming.

Our result showed for the first time that SIRT1 over-expression promoted iPSCs formation by 10–15 folds during reprogramming. The treatment is more potent than RSV (∼6 folds) treatment, mainly due to the fact that SIRT1 over-expression not only increased the expression of *Nanog*, but also reduced *p21* mRNA expression dose dependently. The phenomenon was in line with previous finding that over-expression of SIRT1 strongly attenuated the expression of p53 transcription-dependent apoptosis targets p21 in cancer cells [47]. On the other hand, we found that SIRT1 over-expression had no effect on the expression of mesenchymal-to-epithelial transition (MET) markers, *Snail2* (mesenchymal) and *Cdh1* (epithelial), indicating that SIRT1 may not contribute to MET in the early phase of reprogramming [38].

Based on gene expression profile analyses of our secondary fibroblasts, the secondary MEF reprogramming process could be divided into 3 phases: initiation, maturation and stabilization [38]. In the initiation phase (∼Day 1–5 post-DOX treatment), DOX removal reverts the transcription profile back to the non-DOX treated basal state. This phase is marked by MET, which is a critical event during reprogramming. In the maturation phase (∼Day 6-15), the cells become independent of exogenous MKOS and a subset of pluripotency-associated genes (e.g. Nanog and Sall4) are induced. In the stabilization phase (>Day 16), refinement of the cellular signature and the expressions of other pluripotent markers occur [38]. The effects of RSV and SIRT1 siRNA were most prominent in the initiation phase of reprogramming. Inhibition of the p53/p21 pathway increases the kinetics of iPSC formation by enhancing cell division [48] and abrogation of apoptosis at the onset of iPSC formation [49]. The reduction in *Sirt1* level during the initiation phase (Fig. 2A) may imply less p53 inactivation by SIRT1 deacetylation at this period. So, SIRT1 over-expression or activation at this period could have rescued the cells undergoing stressful reprogramming.

It has been shown that transcription factors-induced iPSCs possess an epigenetic memory of their somatic cells origin [50], [51], and treatment of these cells with chromatin-modifying compounds revert them to fully reprogrammed cells that stably express pluripotent markers, show an indistinguishable epigenetic pattern with ESCs and are able to form chimeras. [50]. RSV was most effective when the treatment covered the whole reprogramming process. Together with ChIP analysis in other study showing that SIRT1 preferentially binds to the promoters of genes that are related to the developmental process in human and mouse ESCs [17], we postulated that SIRT1 may function as an epigenetic regulator modulating the gene expression in the early phases to facilitate reprogramming, possibly through deacetylating lineage-related factors.

The reprogramming efficiency of miPSCs can be enhanced by ESC specific miRNAs [20], [21]. Several miRNAs down-regulate SIRT1 expression in mESCs [22]. MiR-34a is a downstream effector of p53 [52]. In cancer cell lines, miR-34a inhibited Sirt1 expression, leading to an increase in p53 activity and apoptosis [29]. Our observations that miR-34a precursor down-regulated while miR-34a inhibitor up-regulated SIRT1 protein expressions support that *Sirt1* is also a direct target of miR-34a in ESCs. Recently, miR-34a has been shown to be a barrier to reprogramming partly by repression of pluripotency marker genes, including Nanog. Its expression was significantly up-regulated 3 days after reprogramming [53]. Interestingly, our data demonstrated a down-regulation of *Sirt1* in the 2°F/1B MEF within the same period of reprogramming, which might be attributed to the Yamanaka factors-induced miR-34a up-regulation [53]. These observations suggest the involvement of a miR-34a-SIRT1-p53 pathway during reprogramming of MEF. Therefore, we postulate that the induced miR-34a during the early phase of reprogramming may suppress SIRT1 expression, leading to its abrogation on p53 inactivation, and subsequently affecting the reprogramming of mouse fibroblasts.

The postulate is supported by two observations. First, miR-34a precursor inhibited while miR-34a inhibitor and RSV stimulated iPSC formation in primary and secondary MEFs. A higher reprogramming efficiency in the secondary MEFs than the primary MEFs with randomly transfected reprogramming factors [54] is expected because of lower percentage of cells carrying the transgenes in the primary system. Second, tenovin-6, a small molecule that inhibited SIRT1 by suppressing its deacetylation activity of p53 [55], activated p53 and suppressed iPSC formation in the primary system.

In conclusion, SIRT1 expression is closely correlated with the differentiation of ESCs and reprogramming of MEFs. SIRT1 over-expression and SIRT1 activator, RSV promote, while SIRT1 knockdown inhibits iPSCs formation. Such action of SIRT1 is most potent in the initial phase of reprogramming. SIRT1 acts in part through deacetylation of p53, inhibition of p21 and enhancement of Nanog expression. On the other hand, miR-34a forced expression suppresses reprogramming by suppressing SIRT1 expression leading to higher p53 activity. These data together with the stimulatory action of p53 on miR-34a expression in human ESCs [56] supported the operation of a miR-34a-SIRT1-p53 loop (Schematic diagram Fig. 8) during early phase of reprogramming. To our knowledge, this is the first study showing the role of SIRT1 in the reprogramming process. As prolonged suppression of p53 may lead to the formation of iPSCs with DNA lesions and chromosomal aberrations, a transient suppression of the loop at the initiation phase may be a good compromise in this respect as the administration of RSV and SIRT1 siRNA in the initiation phase are most effective in enhancing reprogramming.

## Supporting Information

Figure S1 The relative *NANOG*, *OCT4* and *TP63* or *KRT7* mRNA expression in H9 after induced differentiation with RA (A) and BMP4 (B); The time dependent mRNA expressions of three germ layer markers, *AMY* (C), *REN* (D) and *NEFH* (E) and pluripotent markers, *NANOG* (F) and *OCT4* (G) on Day 4, 8, 12, 16, 20 and 24 during hEB formation; The time dependent mRNA expressions of *TP63* (H), *NANOG* (I) and *OCT4* (J) in H9 after treatment with RA for 3, 5, 7, 9, 11, 13 and 15 days. D0 is the undifferentiated control. The relative *NANOG* (K) and *OCT4* (L) mRNA expressions in H9 after transfected with control-siRNA or Sirt1-siRNA.
(TIF)

Figure S2 Immunocytochemistry of mESC pluripotent cell marker SSEA-1(red) and NANOG (red) in the iPSC colonies formed upon 15 days DOX treatment in 2°F/1B MEF. Green fluorescent indicated the GFP signal.
(TIF)

Figure S3 (A) Western blotting showing the over-expression of SIRT1 protein levels after transfection of 8 and 16 ng/ml SIRT1 plasmids. (B) The relative proliferation rate of MEF after transfection of 4, 8 or 16 ng/ml SIRT1 plasmid.
(TIF)

Figure S4 Western blotting showing acetylated p53, p53 and PCNA upon treatment with 1 and 5 µM tenovin-6.
(TIF)
