# Supplementary figures and images for: Sirtuin 1 Facilitates Generation of Induced Pluripotent Stem Cells from Mouse Embryonic Fibroblasts through the miR-34a and p53 Pathways
Source: PLoS One. 2012 Sep 21;7(9):e45633. doi: 10.1371/journal.pone.0045633 (PMC3448677; doi:10.1371/journal.pone.0045633)

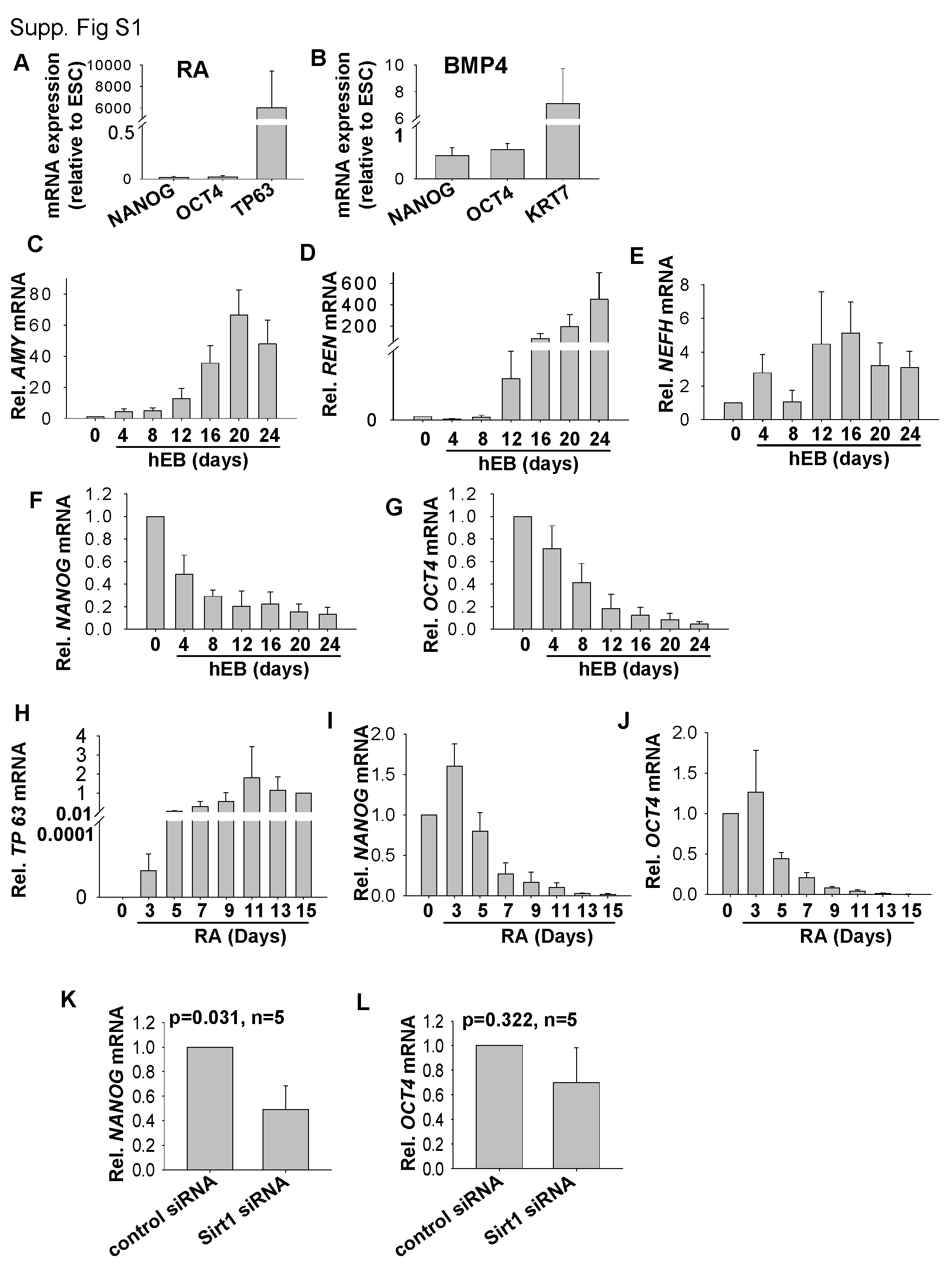

Supplement: Figure S1 — The relative NANOG, OCT4 and TP63 or KRT7 mRNA expression in H9 after induced differentiation with RA (A) and BMP4 (B); The time dependent mRNA expressions of three germ layer markers, AMY (C), REN (D) and NEFH (E) and pluripotent markers, NANOG (F) and OCT4 (G) on Day 4, 8, 12, 16, 20 and 24 during hEB formation; The time dependent mRNA expressions of TP63 (H), NANOG (I) and OCT4 (J) in H9 after treatment with RA for 3, 5, 7, 9, 11, 13 and 15 days. D0 is the undifferentiated control. The relative NANOG (K) and OCT4 (L) mRNA expressions in H9 after transfected with control-siRNA or Sirt1-siRNA. (TIF) [file pone.0045633.s001.tif]

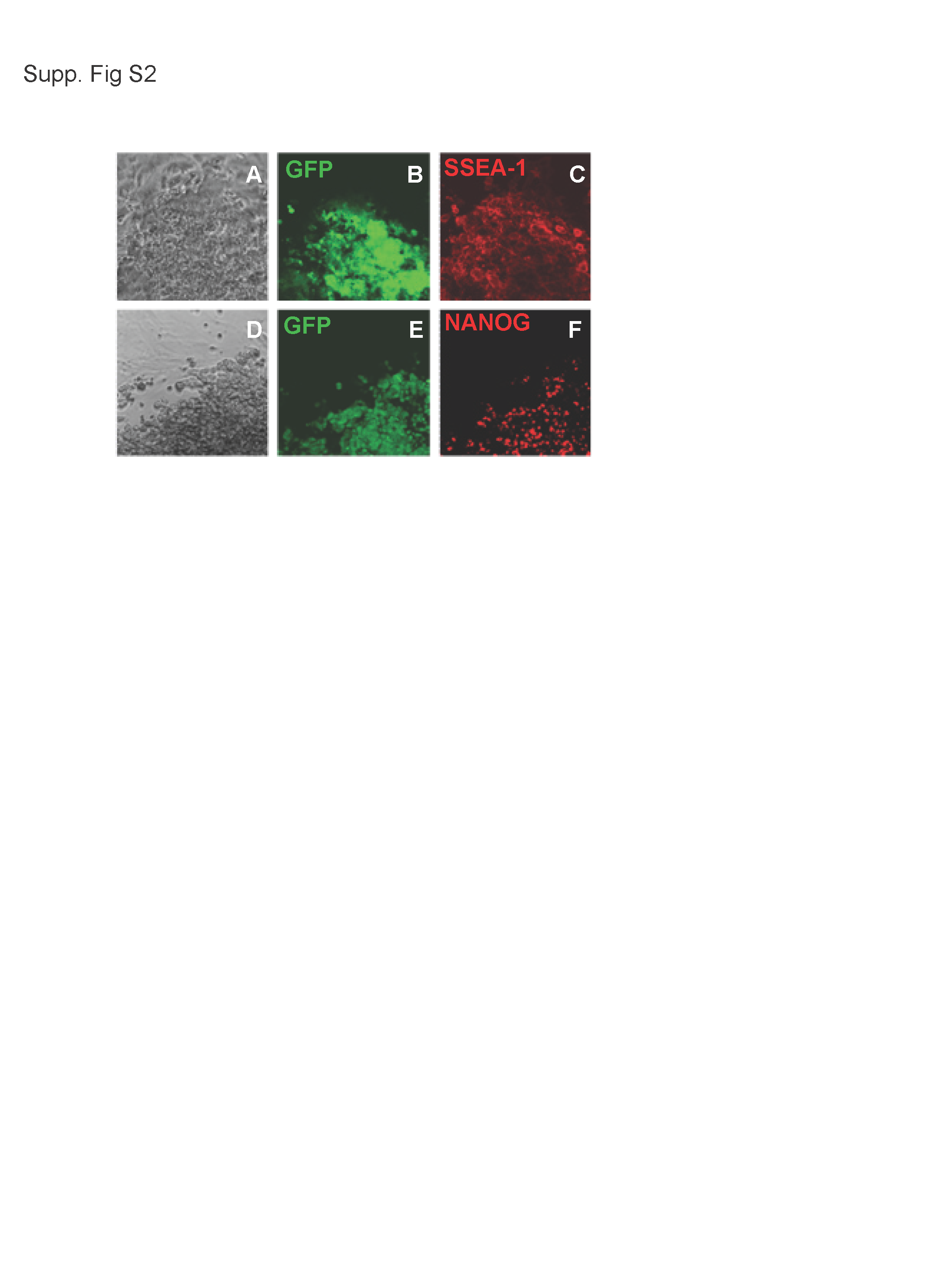

Supplement: Figure S2 — Immunocytochemistry of mESC pluripotent cell marker SSEA-1(red) and NANOG (red) in the iPSC colonies formed upon 15 days DOX treatment in 2°F/1B MEF. Green fluorescent indicated the GFP signal. (TIF) [file pone.0045633.s002.tif]

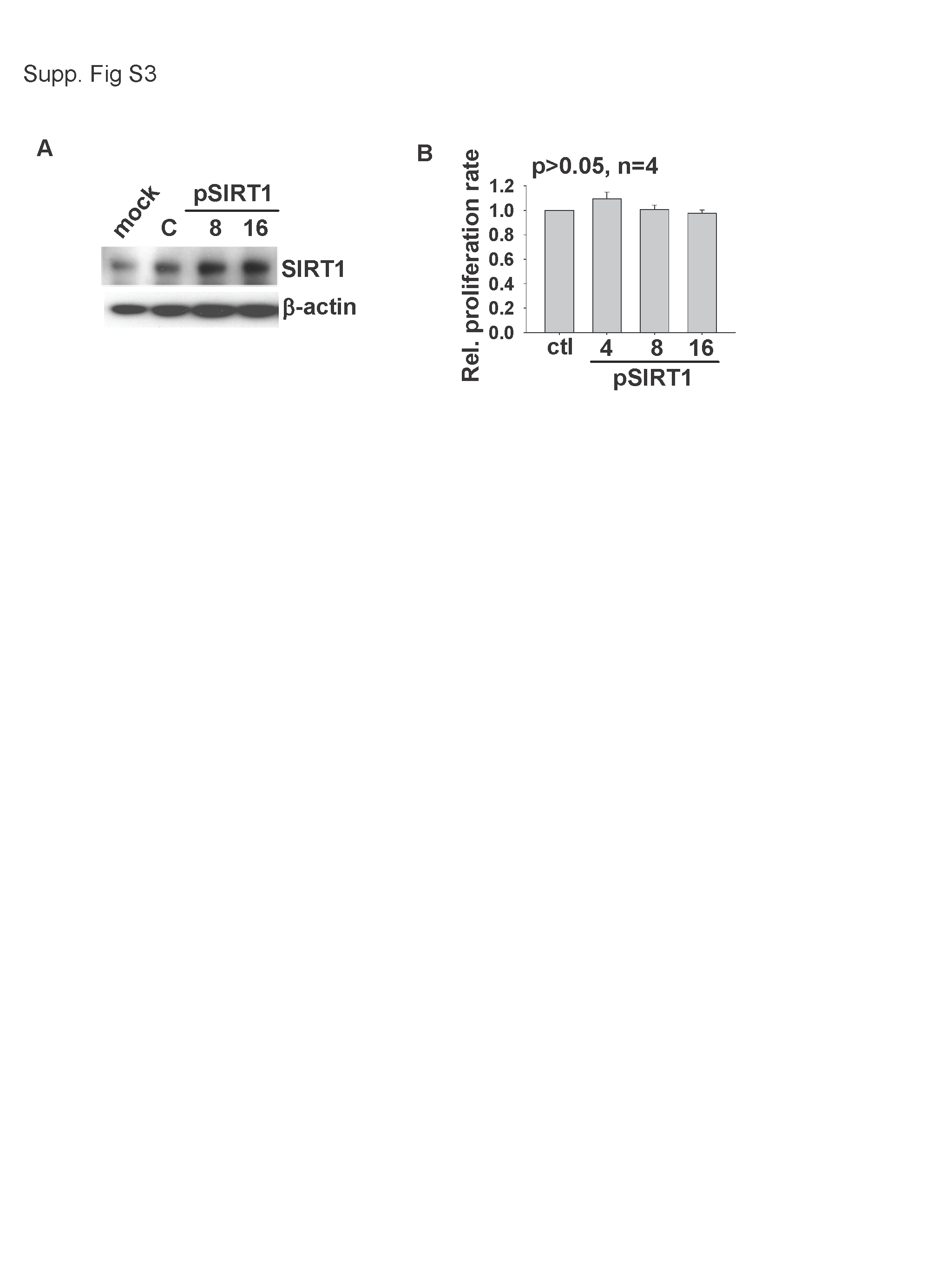

Supplement: Figure S3 — (A) Western blotting showing the over-expression of SIRT1 protein levels after transfection of 8 and 16 ng/ml SIRT1 plasmids. (B) The relative proliferation rate of MEF after transfection of 4, 8 or 16 ng/ml SIRT1 plasmid. (TIF) [file pone.0045633.s003.tif]

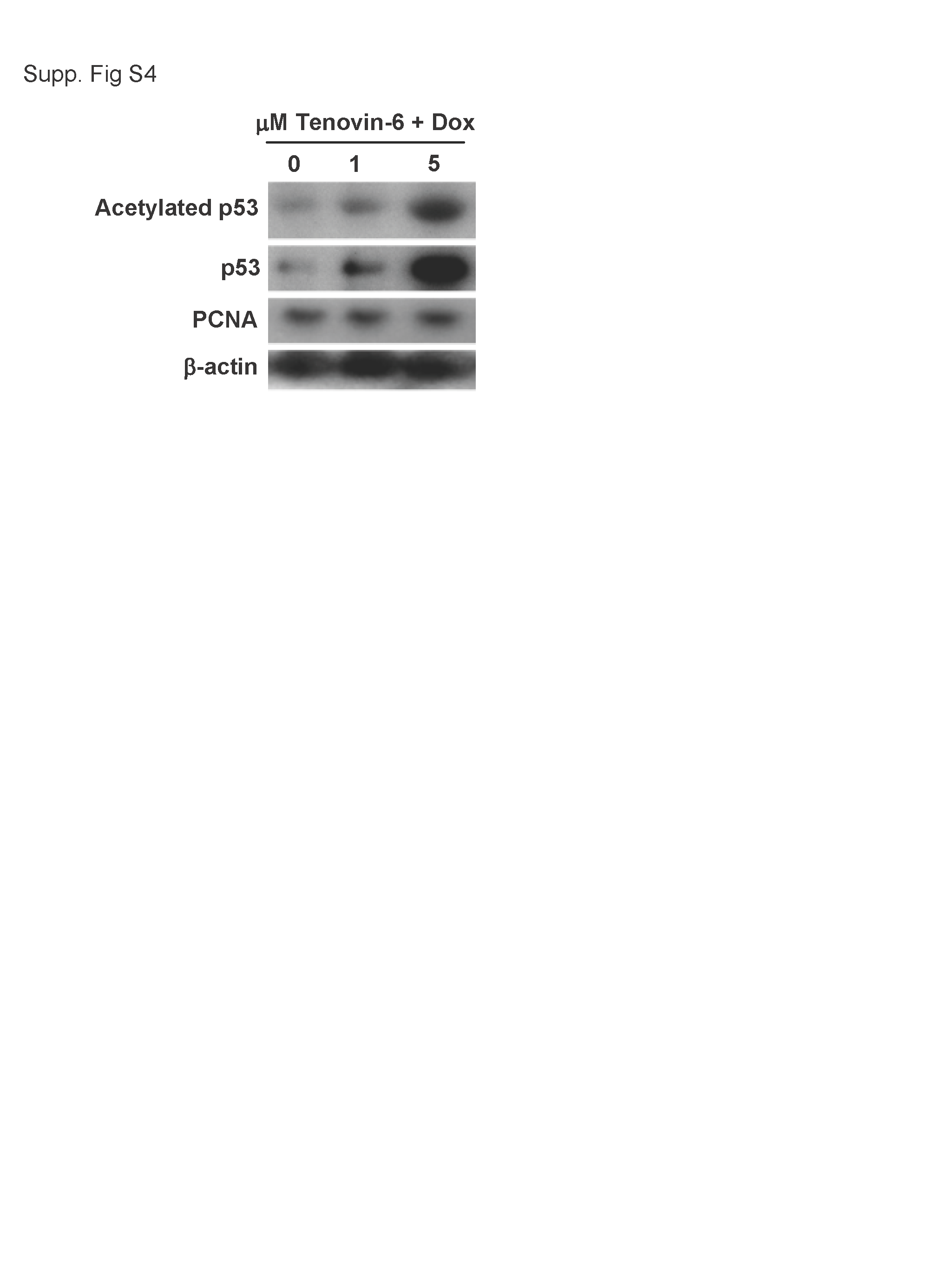

Supplement: Figure S4 — Western blotting showing acetylated p53, p53 and PCNA upon treatment with 1 and 5 µM tenovin-6. (TIF) [file pone.0045633.s004.tif]
